# Mitochondrial Haplogroups Modify the Risk of Developing Hypertrophic Cardiomyopathy in a Danish Population

**DOI:** 10.1371/journal.pone.0071904

**Published:** 2013-08-05

**Authors:** Christian M. Hagen, Frederik H. Aidt, Paula L. Hedley, Morten K. Jensen, Ole Havndrup, Jørgen K. Kanters, Johanna C. Moolman-Smook, Severin O. Larsen, Henning Bundgaard, Michael Christiansen

**Affiliations:** 1 Department of Clinical Biochemistry and Immunology, Statens Serum Institut, Copenhagen, Denmark; 2 Department of Biomedical Sciences, University of Copenhagen, Copenhagen, Denmark; 3 Department of Biomedical Sciences, Stellenbosch University, Cape Town, South Africa; 4 Department of Medicine B, Heart Center, Righospitalet, Copenhagen, Denmark; 5 Department of Cardiology, Roskilde Hospital, Roskilde, Denmark; 6 Department of Cardiology, Gentofte University Hospital, Copenhagen, Denmark; Leibniz-Institute for Arteriosclerosis Research at the University Muenster, Germany

## Abstract

Hypertrophic cardiomyopathy (HCM) is a genetic disorder caused by mutations in genes coding for proteins involved in sarcomere function. The disease is associated with mitochondrial dysfunction. Evolutionarily developed variation in mitochondrial DNA (mtDNA), defining mtDNA haplogroups and haplogroup clusters, is associated with functional differences in mitochondrial function and susceptibility to various diseases, including ischemic cardiomyopathy. We hypothesized that mtDNA haplogroups, in particular H, J and K, might modify disease susceptibility to HCM. Mitochondrial DNA, isolated from blood, was sequenced and haplogroups identified in 91 probands with HCM. The association with HCM was ascertained using two Danish control populations. Haplogroup H was more prevalent in HCM patients, 60% versus 46% (p = 0.006) and 41% (p = 0.003), in the two control populations. Haplogroup J was less prevalent, 3% vs. 12.4% (p = 0.017) and 9.1%, (p = 0.06). Likewise, the UK haplogroup cluster was less prevalent in HCM, 11% vs. 22.1% (p = 0.02) and 22.8% (p = 0.04). These results indicate that haplogroup H constitutes a susceptibility factor and that haplogroup J and haplogroup cluster UK are protective factors in the development of HCM. Thus, constitutive differences in mitochondrial function may influence the occurrence and clinical presentation of HCM. This could explain some of the phenotypic variability in HCM. The fact that haplogroup H and J are also modifying factors in ischemic cardiomyopathy suggests that mtDNA haplotypes may be of significance in determining whether a physiological hypertrophy develops into myopathy. mtDNA haplotypes may have the potential of becoming significant biomarkers in cardiomyopathy.

## Introduction

Mitochondria are responsible for a diversity of essential functions in cardiomyocytes including the production of ATP by oxidative phosphorylation (OXPHOS), several signaling pathways, control of apoptosis, as well as control of the cytosolic calcium concentration [1]. Mitochondria contain 3-10 16,570 bp circular DNA molecules, mitochondrial DNA (mtDNA), comprising a non-coding control region and a coding region containing 37 genes. The genes encode 13 polypeptide components, constituting a small fraction of the many proteins involved in the OXPHOS system, consisting of complex I-IV and complex V (ATP synthase). The rest of the genes code for two species of ribosomal RNA (rRNAs) and 22 species of transfer RNAs (tRNA) required for intra-mitochondrial translation.

**Figure 1:**
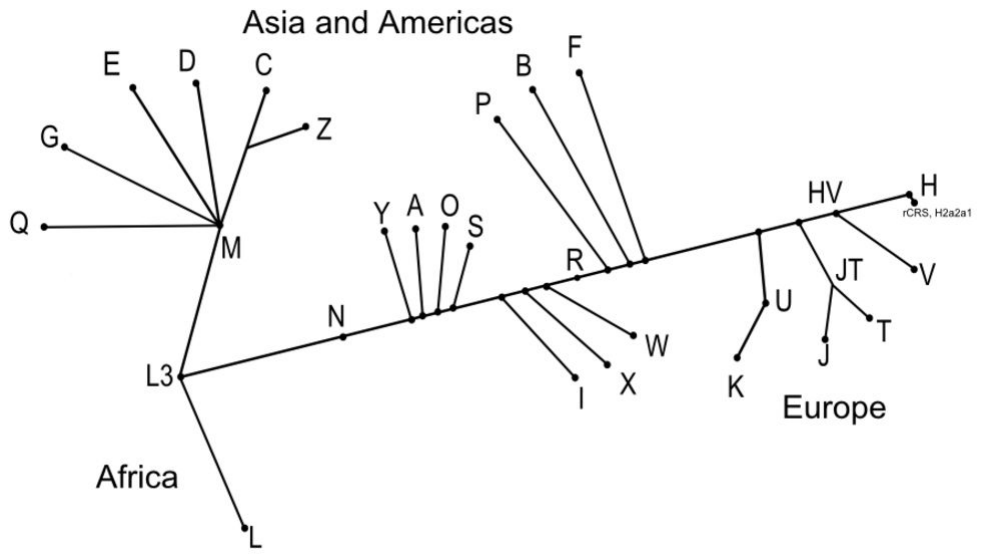
Simplified mtDNA tree based on PhyloTree mtDNA tree Build 15 (30 Sep 2012) (www.phylotree.org).

During evolution, mutations have accumulated sequentially along maternal mtDNA lineages forming groups of related mtDNA genotypes, haplogroups which tend to be regionally and ethnically specific [2] (Figure 1). The haplogroup defining variants encompass transitions and transversions in both protein- and RNA-coding genes (Tables 1 and 2), hence, mtDNA haplogroups are likely to confer different functional characteristics. Indeed, mtDNA haplogroup H has been shown to exhibit differences in VO_2max_ and reactive oxygen species (ROS) damage [3], and H, J and K to confer differences in penetrance or expression of Leber Hereditary Optic Neuropathy [4]. Likewise, haplogroup J, U and H has been associated with reduced and increased risk of Alzheimer and Parkinson disease [5,6] and haplogroup K with reduced risk of in transient ischaemic attack and ischaemic stroke [7]. In addition, haplogroup H and J are associated with increased and reduced of developing ischemic cardiomyopathy, respectively [8].

**Table 1:** Table of haplogroup defining variants used by Benn et al. for mtDNA haplotyping.

Coding-region mtDNA haplogroup markers used by Benn et al.						
Haplogroup:	mt. 7028	mt. 10398	mt. 11719	mt. 12308	mt. 12612	mt.15607
H	C	A	G	A	A	A
HV	T	A	G	A	A	A
K	T	G	A	G	A	A
U	T	A	A	G	A	A
W/I	T	A	A	A	A	A
T	T	A	A	A	A	G
J	T	A	A	A	G	A

**Table 2:** Table of haplogroup defining variants used in this study for mtDNA haplotyping.

mtDNA haplogroup markers used in this study.						
Haplogroup:	Diagnostic SNP:					
H	7028C	2706A	14766C			
U	11467G	12308G				
T	11251G	15607G	4917G			
K	11467G	12308G	10550G	11299C		
J	11251G	13708A	15452A	10398G		
I	10034C	9548G				
W	10034T	11947G				
X	10034T	11947A	6371T			
HV	14766C	11719G				
V	4580A					
D	5178A	16362C				
E	13626T					
N	8701A	10873T				
L3	1018G	10400C	10873C			
R	12705C	14766T	11251A	8281-8289d!	15607A	11467A

!:8281-8289 not present.

The heart has the highest density of mitochondria and the highest oxygen uptake rate of human organs [9-11], consequently, functional differences in mitochondria must be expected to play a more prominent role in defining susceptibility to cardiac disease than to diseases in other organs. This is clearly supported by the many mitochondrial diseases, where cardiomyopathy is part of the clinical syndrome [1] and the finding of mitochondrial dysfunction in HCM [12]. HCM is the most common inherited cardiac disease with a prevalence of 1 in 500 individuals in the general population [13]. In adult patients, 50-60% of all cases are associated with mutations in genes coding for proteins involved in sarcomere function [14]. The disease exhibits a considerable intra- and inter-familial variability with respect to the clinical presentation, some affected present with syncope, dyspnea, angina and palpitations, whilst others are asymptomatic. HCM is a common cause of sudden cardiac death in young adults and athletes [15]. Recently, a study found that haplogroup T might be a risk factor for developing HCM in the Spanish population [16]. Mutations in mtDNA have been found to be frequently occurring (>1:200) in unselected neonates [17] and some of the mutations found have previously been found associated with HCM [18]. These findings indicate that mtDNA haplogroups, in particular J, K, U and H, might play a role as susceptibility factors or modifiers for HCM.

In the present study we examined whether the distribution of mtDNA haplogroups and haplogroup clusters (U+K, J+T, I+W+X and H+V) differed between a consecutively collected Danish HCM cohort and the normal population. We found that haplogroup H and haplogroup cluster HV were susceptibility factors with respect to developing HCM, whereas haplogroup J and K as well as haplogroup cluster UK were protective factors.

## Materials and Methods

### Ethics

All patients gave written informed consent and the study was approved by the ethics committee of Copenhagen and Frederiksberg (KF V92213).

### Patients

Ninety-one unrelated consecutively diagnosed HCM patients identified at, or referred to, Copenhagen University Hospital, Rigshospitalet, Copenhagen, Denmark were included in the study. Patients were subjected to a full clinical evaluation including family history, physical examination, echocardiography and ECG. All fulfilled classical diagnostic criteria for familial HCM [19,20]. The mean age of index patients was 49 years, 62% were male, and 48% of cases were familial. A summary of demographic and clinical characteristics of the patient cohort is given in Table 3. Of the patients, 92% had septal hypertrophy, 6% apical hypertrophy and 2% mid-ventricular hypertrophy. All patients had been screened for mutations in the coding regions of *MYH7, MYBPC3, TTNT2, TPM1, TNNI3, MYL3, MYL2, ACTC, TCAP, CSRP3, CRYAB ,KCNE1-5, GLA* and exons 3, 7, 14, 18, and 49 of *TTN*, as detailed in previous studies [14,21,22]. In 32 index patients putative disease-causing mutations were identified, i.e. 12 in *MYH7*, 8 in *MYBPC3*, 2 in each of *TNNT2, TNNI3* and *GLA*, 1 in each of *ACTC, TPM1, MYL3* and *MYL2*. Two patients were carriers of mutations in both *MYL2* and *MYH7.*

**Table 3:** Demographic and clinical characteristics of HCM probands.

Parameter	Value
Age (years)*	**49 (16)**
**Male/female (ratio)**	**54/37**
BP systolic (mmHg)*	**128 (21)**
BP diastolic (mmHg)*	**76 (14)**
LA (mm)*1	**45 (9)**
Max LVD (mm)*^1^	**20 (6)**
MaxIVS(mm)*^1^	**20 (5)**

* Mean (SD). ^1^ In index patients > 18 years of age. BP, blood pressure; LA, left atrial diameter; MaxLVD, maximal left ventricular wall thickness: MaxIVS, maximal interventricular wall thickness.

### mtDNA haplotyping

DNA was extracted from blood using the Maxwell® 16 System (*Promega, Madison, USA*). An annealing temperature of 60° C was used in all PCR. The PCR products were sequenced using BigDye Terminator v 1.1 Cycle Resequencing (*ABI*), and analyzed on an ABI3730 DNA Analyzer. Primer sequences are available on request. The resulting sequences were compared to the Revised Cambridge sequence (rCRS, GenBank ID:NC_012920) using Sequencher 4.8 software (*Gene Codes, Ann Arbor, USA*). The haplogroups were classified according to PhyloTree mtDNA tree Build 15 (30 Sep 2012) (www.phylotree.org) using diagnostic variants presented in Table 2.

### Population stratification testing

Allele frequencies for the 15 short tandem repeats (STR) loci D8S1179, D21S11, D7S820, CSF1PO, D3S1358, TH01, D13S317, D16S539, D2S1338, D19S433, vWA, TPOX, D18S51, D5S818 and FGA, part of the AmpFLSTR® Identifiler® PCR Amplification Kit (Applied Biosystems, USA) were determined in a sample of 91 unrelated Danish HCM patients (54 H-haplotype carriers) and 90 control samples. The PCR products were separated by capillary electrophoresis on an ABI3730 DNA Analyzer (Applied Biosystems). Allele designations were determined by comparison with the GeneScan™–500 LIZ® Size Standard (Applied Biosystems) using GeneMarker v 2.4.0 (SoftGenetics, USA). Allele frequencies, Hardy–Weinberg equilibrium test (100,000 steps in Markov chain with 10,000 of dememorization steps) and to get a measure for genetic differentiation pairwise fixation index F_ST_ were calculated with GenePop v 4.2 [23]. For the purposes of determining whether the H-haplotype carriers were a distinct genetic group; the statistical analyses assumed the H-haplotype carriers and non-H-haplotype carriers were two different populations.

### Statistical analysis

Populations were compared using Fisher’s exact test. Odds-ratios (OR) and their confidence intervals (CI) were calculated. A significance level of 5% was considered significant. Each haplogroup and clusters (H+V, T+J, U+K, I+W+X) were compared to all other haplogroups pooled in to a single group. Evolutionary closely related haplogroups W and I, which have low frequencies was pooled into a single group. Statistical analysis was performed using Stata version 12.1 (*StataCorp LP, College Station, Texas*).

## Results

The mtDNA haplogroup distribution of the 91 HCM patients is shown in Table 4. Also shown are the reported haplogroup distributions in a large Danish population (n= 9254) study by Benn et al. [24], performed in the same geographic location of Copenhagen as the hospital where the HCM patients, participating in this study, were recruited, and in a smaller Danish study (n=201) performed as a control study for forensic examinations by Mikkelsen et al. [25], Not all haplogroups were identified in the control population studies. MtDNA haplotyping performed by Mikkelsen et al. were based on variants in the hyper-variable regions 1 and 2 according to [26] and the mtDNA haplotying performed by Benn et al. were based on haplogroup specific markers in the coding region of the mtDNA (Table 2).

**Table 4:** Distribution of haplogroups in the HCM cohort and two control populations.

Haplogroup	HCM cohort (n=91).	Benn et al. 2008 (n=9254)	Mikkelsen et al. 2010 (n=201)
H	60,4 (55)	45,9 (4244)	41,1 (83)
U	9,9 (9)	15,9 (1469)	12,9 (26)
T	13,2 (12)	9,9 (912)	8,4 (17)
J	3,3 (3)	9,1 (843)	12,4 (25)
I	1,1 (1)	3,8^1^ (350)	3 (6)
W	0	3,8^1^ (350)	0,5 (1)
X	2,2 (2)	n.d	1 (2)
K	2,2 (2)	6,2 (571)	9,9 (20)
HV	1,1 (1)	4,5^2^ (412)	3,5 (7)
V	4,4 (4)	4,5^2^ (412)	n.d
Z	0	3,5 (324)	n.d
N	0	n.d	1
L3	0	n.d	0,5 (1)
R	0	n.d	5,9 (12)
D	1.1(1)	n.d	n.d
E	1.1 (1)	n.d	n.d

^1^ **Haplogroup was not distinguished;**. **^2^ Haplogroup HV and V were not distinguished.**

All major haplogroups were identified in this study and compared with the haplogroup frequencies obtained from two studies by Benn et al. and Mikkelsen et al.

Haplogroup H was found to have a higher frequency in HCM probands, 60% versus 41%(p = 0.006), and 46%, (p = 0.003) as compared to controls (Table 4). This difference translates into odds-ratios (OR) for having HCM of 1.8 (CI = [1.16-2.83]) and 2.17 (CI = [1.27-3.72]) (Tables 5 & 6), respectively. On the contrary, the prevalence of haplogroup J was significantly lower in HCM probands, 3.3% vs. 12.4% (p = 0.017) (Table 5) when compared to the forensic control population, yielding an OR=0.24, CI= [0.05-0.82] (Table 6). The difference in prevalence of haplogroup J was only borderline significant when the HCM group was compared with the population controls, 3.3% vs. 9.1% (p = 0.06) (Table 5) yielding an, OR = 0.34, CI = [0.07-1.03]) (Table 6). These results indicate that haplogroup H acts as a susceptibility factor and haplogroup J as a protective factor for HCM.

**Table 5:** Odds-ratios for HCM of haplogroups or combinations of haplogroups compared to Benn et al. 2008.

HCM-cohort vs. Benn et al.	Test setup.	Fisher’s exact test P-value	Odds-ratio	95% Confidence Interval
Haplogroups compared	H vs. Non-H	0,006	1,8	1,16-2,83
	U vs. Non-U	0,147	0,58	0,26-1,16
	T vs. Non-T	0,288	1,39	0,69-2,58
	J vs. Non-J	0,063	0,34	0,07-1,03
	I+W vs. Non-I+W	0,265	0,28	0,007-1,62
	K vs. Non-K	0,181	0,34	0,04-1,28
Cluster comparison.	HV vs. Non-HV	0.003	1.91	1.22-3.07
	UK vs. Non-UK	0.021	0.49	0.23-0.92
	JT vs. Non-JT	0.683	0.84	0.45-1.48

**Table 6:** Odds-ratios for HCM of haplogroups or combinations of haplogroups compared to Mikkelsen et al. 2008.

HCM-cohort vs. Mikkelsen et al.	Test setup.	Fisher’s exact test P-value	Odds-ratio	95% Confidence Interval
Haplogroups compared	H vs. Non-H	0,003	2,17	1,27-3,72
	U vs. Non-U	0,561	0,74	0,29-1,71
	T vs. Non-T	0,212	1,64	0,68-3,84
	J vs. Non-J	0,017	0,24	0,05-0.82
	I vs. Non-I	0,442	0,36	0,007-3.05
	K vs. Non-K	0,018	0,20	0,02-0.87
	X vs. Non-X	0,605	2.23	0.16-31.2
Cluster comparison	UK vs. Non-UK	0.038	0.46	0.21-0.97
	JT vs. Non-JT	0.43	0.75	0.36-1.48
	IWX vs. Non-IWX	0.76	0.72	0.12-3.0

Comparing the prevalence of haplogroup K in the HCM group with that of the two control populations disclosed a significant difference (Table 4) with the forensic control group, p = 0.018 and an OR for HCM of 0.20, CI = [0.02-0.87] (Table 6). However, the difference was not significant when compared with the population based control population, p =0.181 and an OR= 0.34, CI= [0.04-1.28] (Table 5). None of the other haplogroup frequencies differed significantly between the HCM and control groups (Tables 5 and 6).

When comparing the prevalence of the evolutionarily related haplogroups (HV, JT, KU and IWX) (Figure 1), we found that the prevalence of the JT cluster was not significantly different from that of the control populations (Tables 5 and 6), indicating that the haplogroup defining variants distinguishing haplogroup J and T could explain the protective effect of haplogroup J. The prevalence of the UK cluster was found to be significantly reduced compared to both population controls (p = 0.02, OR = 0.49, CI= [0.23-0.92]) and forensic controls (p = 0.038, OR = 0.46, CI = [0.21-0.97]). This suggests that the UK cluster is a protective factor against the development of HCM.

The prevalence of the HV cluster was found to be significantly increased in HCM probands when compared to the population based control group, p = 0.003, OR = 1.91, CI = [1.22-3.07]. Haplogroup V was not identified in the forensic control material precluding a comparison with that group.

There was no appreciable difference with respect to age, gender distribution, frequency of sarcomere mutations identified and left ventricle dimensions as a function of haplogroup when the HCM group was broken down with respect to mtDNA haplogroups (Table 7).

**Table 7:** Clinical characteristics and mutation occurrence as a function of haplogroup.

Haplogroup (n)	H (55)	T(12)	J(3)	K(2)	U(9)	Rest (10)
Median age (range)	51 (5-77)	43.3 (8-71)	38 (19-45)	44.5 (43-46)	53 (39-74)	66.5 (14-76)
Mean MaxLVD (SD)	21.3 (5.2)	21.8 (4.1)	22.7 (8.4)	22.0 (2.0)	21.3 (3.2)	17.4 (3.9)
Male/female-ratio	33/22	7/5	2/1	2/0	7/2	5/5
Mutations	21	8	1	2	4	4
Mutations (%)	41	66.6	33.3	100	44	40

To test for bias as a result of population stratification all HCM probands and 90 controls were analyzed for 15 short tandem repeats (STR). All STR loci investigated were in Hardy-Weinberg equilibrium, P=0.99 and P=0.83 for HCM and control populations respectively; a pairwise F_ST_ value of 0.003 indicates that there is no difference between the HCM population and the control population. Similarly, an F_ST_ of 0.001 indicates that there are no differences between the H-haplotype carrying HCM population and the non-H-haplotype carrying HCM population, indicating that the H-haplotype carrying population of HCM probands investigated here is not stratified. The HCM population is as mixed as the background population.

## Discussion

Here we have shown that mtDNA haplogroup H and haplogroup cluster HV constitute susceptibility factors and haplogroup J and the UK cluster constitute protective factors for HCM in a Danish population. Our finding, that haplogroup H is a risk factor and J a protective factor is consistent with the recent finding that haplogroup H is a risk factor and J a protective factor for ischemic cardiomyopathy where ROS has the same putative etiological role [8].

The STR analysis showed that the association between haplogroup H and HCM is not a result of population stratification.

Haplogroup H is defined by the non-synonymous variant, m.14766C, p. MT-CYB: Thr7Ile (also defining HV and V), and the synonymous variant m.7028C in *MT-COI* and the rRNA variant m.2706G in *MT-RNR2*. p. MT-CYB: Thr7Ile is not conserved, CI=1.4% [27], but has been predicted, based on structural analysis, to have impact on the efficiency on ETS [28]. When comparing the haplogroup defining variants of H with the ones of haplogroup J, the differences in non-synonymous variants are, apart from m.C14766C, m.A10398G in *MT-ND3*, m. G13708A in *MT-ND5*, and m. C15452A in *MT-CYB*. These variants result in amino acid substitutions in subunits of Complex I and Complex III, both organized in supercomplexes with Complex IV in the constellation CI_1_CIII_2_CIV_1_ [9,29,30] which are the primary sites of ROS generation. These mtDNA variants may influence the stability and/or activity of the supercomplexes and thereby induce variation in ATP production, electron leakage and/or ROS production [31,32]. As the control populations were not haplotyped to the level of sub-haplotypes, we cannot examine if there are significant differences in the sub-haplotype frequencies. Hence, it was not possible to identify single SNPs that could be responsible for the difference in association with HCM.

Haplogroup H has previously been reported to be physiologically different from other common European haplogroups; Martínez-Redondo et al. [3] found that mitochondria belonging to haplogroup H have higher VO_2max_ and is associated with increased mitochondrial oxidative damage compared to mitochondria of haplogroup J in skeletal muscle. However, the difference disappears with steady exercise [33]. The group suggests that the high VO_2max_ reflects that the electron transport system (ETS) is more tightly coupled in haplogroup H than in haplogroup J mitochondria. Consequently, haplogroup H confers higher ROS production than haplogroup J [3,34].

Altered mitochondrial function is evident in pathological cardiac hypertrophy and ROS is suggested to contribute to this alteration [12]. ROS act as signaling molecules in several pathways, which participate in the control of cell proliferation, differentiation, apoptosis and senescence [10,35,36]. ROS are cytotoxic if not controlled, as the reactive species can modify DNA, oxidate and inactivate iron–sulfur proteins and stimulate lipid peroxidation, causing altered function or degradation of these [1]. Cells have an antioxidant defense compromising several enzymes, such as mitochondrial superoxide dismutase (mtSOD), which maintain balance (redox homeostasis), establishing protection from and control of ROS generation [29]. Oxidative stress is known to cause contractile failure in the myocardium and can induce myocyte hypertrophy, apoptosis, interstitial fibrosis [10,37] and cardiomyopathy [38]. Increased ROS production may lead to myocardial remodeling through activation of matrix metalloproteinases [10,37]. In accordance with the tentative role of increased ROS production as the cause of the haplogroup associated differences in HCM susceptibility, the myocardial ROS level has been found to correlate with the functional reduction of the failing myocardium [39,40].

Interestingly, Benn et al (2008) found no association with over-all morbidity, longevity or ischemic cardiovascular disease and mtDNA haplogroups in the Danish population-based control population [24]. This suggests that the effect of mtDNA haplogroups is a modifier effect requiring the presence of a specific pathological process. This is in agreement with studies on transgenic mouse and rat models that have shown that there is an increased tension-dependent ATP consumption associated with mutations in sacomere genes causing HCM [41]. In thirty-two of the 91 HCM probands analysed here, HCM causing mutations in sarcomere genes had been identified. Such mutations may result in impaired ATP utilization and thereby an increase in the workload of the mitochondria and - concomitantly - increased ROS production. We suggest that the increased baseline ROS production in haplogroup H could lower the threshold of the cardiomyocytes and thereby exacerbate or precipitate HCM. Ascribing ROS as a modifying factor is in concordance with the finding that haplogroup J has a lower OXPHOS coupling, i.e. lower ATP and ROS production, than haplogroup H [3].

Clustering haplogroup UK and comparing with the two control populations indicated that cluster UK is a protective factor for HCM. Recently a study investigating differences in OXPHOS of haplogroup H and K (H1, H5, H13, K1 and K2), using osteosarcoma cybrids have reported that K cybrids had significantly lower mitochondrial inner membrane potential than H, which is in agreement with the significantly lower endogenous leaking and uncoupled respiration found in K per ETS unit compared to H [27]. They found no differences in ROS levels. However, as mentioned by the authors, methods used for ROS measurements are not reliable and this poses a challenge for precise quantitation of ROS [27,42,43]. The authors also mention that the only haplogroup defining non-synonymous SNP (m.14798T>C, p. MT-CYB: Phe18Leu) present in the cybrid cell K is also a marker for haplogroup J1c, and suggests that it is of functional importance because of its conservation and location in cytochrome *b* [27]. Furthermore, both J1c and K are underrepresented in patients with Parkinson’s disease and over-represented in centenarians and in patients with LHON and multiple sclerosis, indicating a similar physiological characteristic of these haplogroups [5,27,44].

Haplogroup T, defined by m.13368A, has previously been associated with an increased risk of developing HCM in a Spanish population [16]. Our study could not confirm this finding, however a trend is present (Tables 5 and 6). The discrepancy could be due to the differences in haplogroup/sub-clade frequencies in the Danish and Spanish population or specific differences that may modify the significance of the modifiers, through variation of other factors, e.g. ethnic background, life style and environmental influences [45,46].

We found no significant differences when comparing the mean age of diagnosis or the number of genetic diagnosed patient between the haplogroups. This supports that the mtDNA haplogroup is not causative of the disease. However, the small number of probands in most of the haplogroups precludes any firm conclusions as to a possible gender-bias or differential clinical characteristics, as previously described for the U haplogroup in Alzheimer’s disease [6]. Even when corrected conservatively for multiple testing using Bonferroni, the association with susceptibility for HCM was significant (p = 0.036 for Benn et al. and p = 0.021 for Mikkelsen et al.) for haplogroup H, whereas the association with other haplogorups where not (data not shown). However the findings are not just a result of multiple testing, but rather based on a hypothesis of experimentally founded functional and clinical significance of mtDNA haplogroups.

In conclusion, our findings indicate that mtDNA haplogroup H and haplogroup cluster HV are susceptibility factors for developing HCM. The opposite is seen for haplogroup J and the UK cluster. As ORs are considerable, i.e. ~ 2, ~ 0.3 and 0.5, respectively, we suggest that mtDNA haplotyping is assessed with respect to its potential as a risk marker for development of HCM in families with sarcomere mutations and in conditions predisposing to HCM of non-genetic etiology, i.e. hypertension, excessive exercise or obesity among others.

### Limitations

The study is limited by the small size, n = 91, of the HCM cohort; on the other hand, it could be considered a strength that a prognostic biomarker has a statistically significant effect in just 91 patients. Furthermore, the associations are largely similar when compared to two different Danish control populations. However, we cannot exclude a skewing in the haplogroup distribution of the cohort, and the associations described should be confirmed in other HCM cohorts. However, the gene distribution of the disease-causing mutations is similar to that seen in other cohorts, suggesting that no skewing is present. This is also experimentally corroborated by the STR analysis. It should also be realized that the mtDNA haplogroups may have geographically distinct genetic backgrounds that may limit the results to – in this case – cohorts of northern-European descent. Finally, a major argument for considering the findings here non-random is the putative functional correlation.
